# Interventions to Improve Adolescent Nutrition: A Systematic Review and Meta-Analysis

**DOI:** 10.1016/j.jadohealth.2016.06.022

**Published:** 2016-10

**Authors:** Rehana A. Salam, Mehar Hooda, Jai K. Das, Ahmed Arshad, Zohra S. Lassi, Philippa Middleton, Zulfiqar A. Bhutta

**Affiliations:** a Division of Women and Child Health, Aga Khan University, Karachi, Pakistan; b Robinson Research Institute, University of Adelaide, Adelaide, Australia; c South Australian Health and Medical Research Institute and The University of Adelaide, Adelaide, Australia; d Centre for Global Child Health, The Hospital for Sick Children, Toronto, Canada; e Center of Excellence in Women and Child Health, The Aga Khan University, Karachi, Pakistan

**Keywords:** Adolescent nutrition, Preconception nutrition, Pregnant adolescents, Micronutrient supplementation

## Abstract

Adequate adolescent nutrition is an important step for optimal growth and development. In this article, we systematically reviewed published studies till December 2014 to ascertain the effectiveness of interventions to improve adolescent nutrition. We found one existing systematic review on interventions to prevent obesity which we updated and conducted de novo reviews for micronutrient supplementation and nutrition interventions for pregnant adolescents. Our review findings suggest that micronutrient supplementation among adolescents (predominantly females) can significantly decrease anemia prevalence (relative risk [RR]: .69; 95% confidence interval [CI]: .62–.76) while interventions to improve nutritional status among “pregnant adolescents” showed statistically significant improved birth weight (standard mean difference: .25; 95% CI: .08–.41), decreased low birth weight (RR: .70; 95% CI: .57–.84), and preterm birth (RR: .73; 95% CI: .57–.95). Interventions to promote nutrition and prevent obesity had a marginal impact on reducing body mass index (standard mean difference: −.08; 95% CI: −.17 to .01). However, these findings should be interpreted with caution due to significant statistical heterogeneity.

Adolescent nutrition is crucial for proper growth and development and a prerequisite for achieving full developmental potential. Suboptimal nutrition may contribute to delayed and stunted growth [1] as well as impaired development. As adolescents undergo a period of rapid growth and development, adequate nutrient intake (of both macro and micronutrients) is critical. Many of the risk factors that impact maternal and newborn health exist right from adolescence, including nutritional deficiencies. Prepregnancy wasting in adolescents is usually reflected as low body mass index (BMI < 18.5). Low BMI significantly increases perinatal risks including stillbirths, preterm births, small for gestational age, and low birth weight (LBW) babies [2]. Iron deficiency anemia is among the top 10 causes of disability-adjusted life years lost among adolescents [2]. Concern is especially warranted for adolescent girls because their iron requirements are relatively high (due to growth spurts, sexual maturation, and menstrual losses) and because they may be on the cusp of motherhood. While most programs are targeted at pregnant women, the depletion of iron stores in women starts during adolescence with the onset of menstruation. More recently, there has been a growing interest in adolescent girls' nutrition as a means to improve the health of women and children. Each year around 16 million babies are born to adolescent girls between the ages of 15 and 19 years, accounting for over 10% of the total births each year [3]. Pregnancy in adolescence is associated with greater risk to the mother and newborn—including anemia, mortality, stillbirths, and prematurity—especially since the adolescent girls are not physically mature themselves [3]. Adolescent girls are two to five times more likely to die from pregnancy-related causes than women aged 20–29 years [3]. Girls younger than 19 years have a 50% increased risk of stillbirths and neonatal deaths, as well as an increased risk for preterm birth, LBW, and asphyxia [3]. These health risks further increase for girls who become pregnant earlier than 15 years and are somewhat reduced for older adolescents aged 18–19 years.

Over the last two decades, increasing rates of overweight and obesity among children and adolescents have been observed in many countries [4], [5]. Many low- and middle-income countries (LMICs) now bear a double burden of nutritional disorders due to the emerging issue of overweight and obesity along with the existing high rates of stunting and other micronutrient deficiencies [6], [7]. Childhood overweight is associated with multiple immediate and long-term risks including raised cholesterol, raised triglycerides, type 2 diabetes, high blood pressure, adult obesity, and its associated consequences [8], [9]. Prepregnancy overweight has been linked to two of the foremost causes of maternal mortality (hypertensive disorders of pregnancy and gestational diabetes mellitus) [10], [11], [12], [13] as well as other adverse pregnancy outcomes, including poor lactation practices [14], [15], obstetric anesthesia–related complications [16], prolonged gestation [17], [18], maternal infectious morbidity [19], and decreased success with trials of labor.

This article is part of a series of reviews conducted to evaluate the effectiveness of potential interventions for adolescent health and well-being. Detailed framework, methodology, and other potential interventions have been discussed in separate articles [20], [21], [22], [23], [24], [25], [26]. In this article, we systematically reviewed published literature to ascertain the effectiveness of interventions to promote nutrition among adolescents comprising of micronutrient supplementation, nutrition interventions for pregnant adolescents, and interventions to prevent obesity.

## Methods

For the purpose of this review, the adolescent population was defined as aged 11–19 years; however, since many studies targeted youth (aged 15–24 years) along with adolescents, exceptions were made to include studies targeting adolescents and youth. Studies were excluded if they targeted age groups other than adolescents and youth or did not report segregated data for the age group of interest. Searches were conducted till December 2014, and we did not apply any limitations on the start search date or geographical settings. Outcomes were not prespecified, and we included all the outcomes reported by the study authors. We searched systematically for existing reviews and took a systematic approach to consolidate the existing evidence through the following methodologies:

1. De novo review: For interventions where no reviews existed, we conducted a new review; and
2. Updating existing reviews: We updated the existing systematic reviews only if the existing review included evidence before 2011.

### Methodology for de novo reviews

For de novo reviews, our priority was to select existing randomized, quasi-randomized and before/after studies, in which the intervention was directed toward the adolescent age group and related to nutritional outcomes. A separate search strategy was developed for each aspect using appropriate keywords, Medical Subject Heading, and free text terms. The following principal sources of electronic reference libraries were searched to access the available data: The Cochrane Library, Medline, PubMed, Popline, LILACS, CINAHL, EMBASE, World Bank's Jolis search engine, CAB Abstracts, British Library for Development Studies at Institute of Development Studies, the World Health Organization regional databases, Google, and Google Scholar. The titles and abstracts of all studies identified were screened independently by two reviewers for relevance and matched. Any disagreements on selection of studies between these two primary abstractors were resolved by the third reviewer. After retrieval of the full texts of all the studies that met the inclusion/exclusion criteria, data from each study were abstracted independently and in duplicate into a standardized form. Quality assessment of the included randomized controlled trials (RCTs) was done according to the Cochrane risk of bias assessment tool. We conducted meta-analysis for individual studies using the software Review Manager, version 5.3 (Cochrane Collaboration, London, United Kingdom). Pooled statistics were reported as the relative risk (RR) for categorical variables and standard mean difference (SMD) for continuous variables between the experimental and control groups with 95% confidence intervals (CIs). A grade of “high,” “moderate,” “low,” and “very low” was used for grading the overall evidence indicating the strength of an effect on specific health outcome according to the Grading of Recommendations Assessment, Development and Evaluation criteria [27].

### Methodology for updated reviews

We updated the existing systematic reviews only if the most recent review on a specific intervention was conducted before December 2011. For updating the existing reviews, we adopted the same methodology and search strategy mentioned in the existing review to update the search and find all the relevant studies after the last search date of the existing review. After retrieval of the full texts of all the articles that met the inclusion/exclusion criteria, data from each study were abstracted independently and in duplicate into a standardized form. Information was extracted on study design, geographical setting, intervention type and description, mode of delivery, and outcomes assessed. We then updated the estimates of reported outcomes by pooling the evidence from the new studies identified in the updated search and reported new effect size for the outcomes of interest with 95% CIs. We then assessed and reported the quality of included reviews using the 11-point assessment of the methodological quality of systematic reviews criteria [28].

## Results

Based on our search results, we updated one systematic review and conducted two de novo reviews.

**Figure 1:**
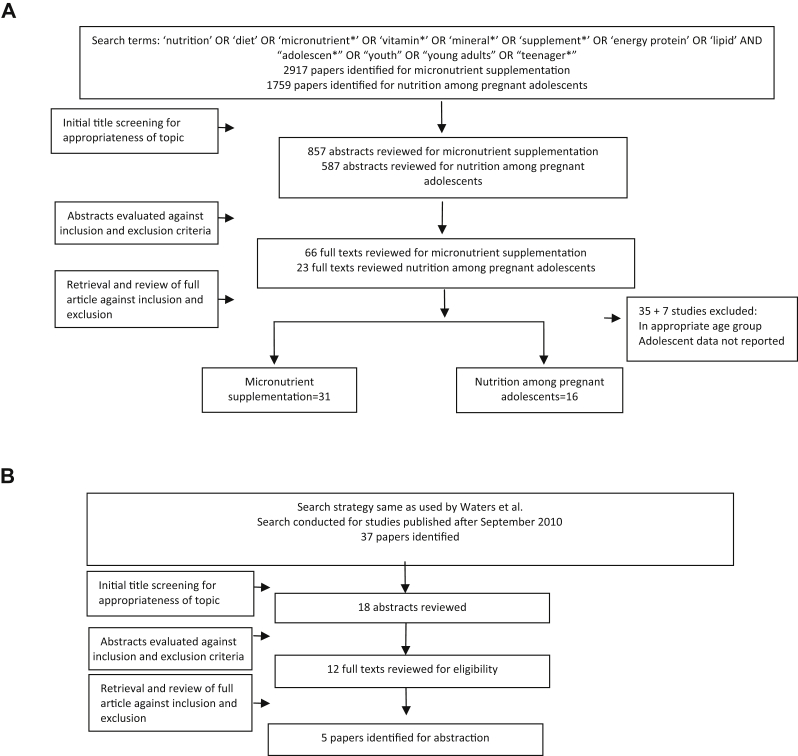
(A) Search flow diagram for de novo reviews (micronutrient supplementation and nutrition for pregnant adolescents). (B) Search flow diagram for review update (interventions to prevent obesity).

For the impact of “micronutrient supplementation among adolescents” and “nutrition interventions for pregnant adolescents,” we conducted de novo reviews (as there were no relevant existing reviews) while for interventions to prevent obesity, we updated an existing Cochrane review by Waters et al. [29]. Figure 1A describes the search flow, and the characteristics of the included studies for the de novo reviews are detailed in Table 1.

The outcome quality for micronutrient supplementation was rated as “moderate quality” because for various outcomes there was considerable heterogeneity; and generalizability was limited to females because most of the studies included female participants. A summary of quality of evidence is provided in Table 2. The quality of outcomes for interventions for pregnant women was rated to be moderate to low due to the study design limitations, heterogeneity, and limited generalizability of the interventions (Table 3). The quality of outcomes for promoting healthy nutrition and preventing obesity was rated to be of “moderate” quality due to design limitation, heterogeneity, and limited generalizability to high-income countries (HICs) only (Table 4).

### Micronutrient supplementation for adolescents

A total of 31 studies were included, of which 23 were conducted in LMICs [30], [31], [32], [33], [34], [35], [36], [37], [38], [39], [40], [41], [42], [43], [44], [45], [46], [47], [48], [49], [50], [51], [52], [53], [54], [55], [56], [57], [58], [59], [76]. Studies evaluated the effectiveness of iron, folic acid, vitamin A, vitamin D, vitamin C, calcium, zinc, and multiple micronutrients supplementation to adolescent population. Thirteen studies evaluated the impact of iron/iron folic acid supplementation alone, nine studies evaluated the impact of iron/iron folic acid in combination with other micronutrients, two studies evaluated the impact of multiple micronutrients alone, two studies evaluated zinc supplementation while five studies supplemented with calcium and vitamin D. The intervention was mostly implemented in schools with the exception of five community-based studies [27], [28], [29], [30], [31]. Most studies evaluated the impact of micronutrient supplementation on adolescent girls except for nine studies that included adolescent boys and girls.

**Figure 2:**
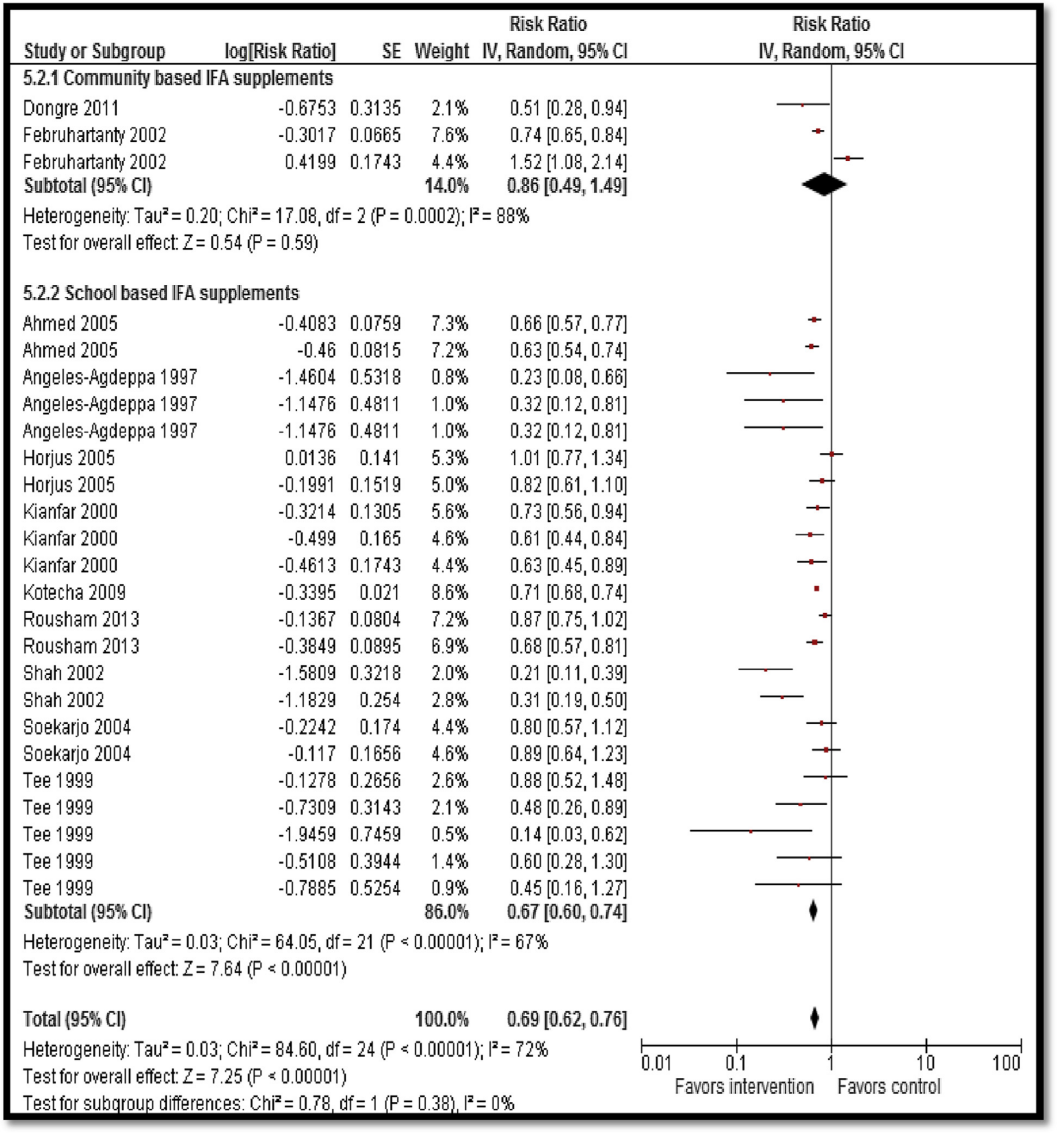
Impact of iron/iron folic acid supplementation on anemia. IFA = iron folic acid; IV = inverse variance; SE = standard error.

Findings from moderate-quality evidence suggest an overall significant reduction in anemia (as defined by study authors) with iron/iron folic acid supplementation alone or in combination with other micronutrient supplementation (RR: .69; 95% CI: .62–.76; Figure 2). Subgroup analysis according to the delivery settings suggests that school-based delivery significantly reduced anemia (RR: .67; 95% CI: .60–.74) while evidence from community-based delivery was underpowered. School-based delivery of iron/iron folic acid supplementation alone or in combination with other micronutrient supplementation was also associated with improved serum hemoglobin (mean difference [MD]: 1.94 g/dl; 95% CI: 1.48–2.41), ferritin (MD: 3.80 mcg/L; 95% CI: 2.00–5.59), and iron (MD: 6.97 μmol/L; 95% CI: .19–13.76). Zinc supplementation led to improved serum zinc concentrations (MD: .96 mcg/dl; 95% CI: .81–1.12) while calcium and vitamin D supplementation did not have a clear impact on vitamin D levels and parathyroid hormone. Gender-specific subgroup analysis suggests significant improvements in both genders; however, most of the studies were conducted on adolescent girls.

### Nutrition interventions among “pregnant adolescents”

**Figure 3:**
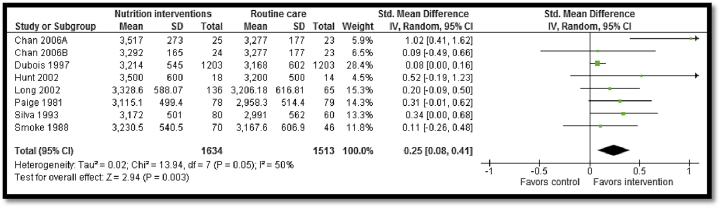
Impact of nutritional interventions for pregnant women on mean birth weight. IV = inverse variance; SD = standard deviation.

**Figure 4:**
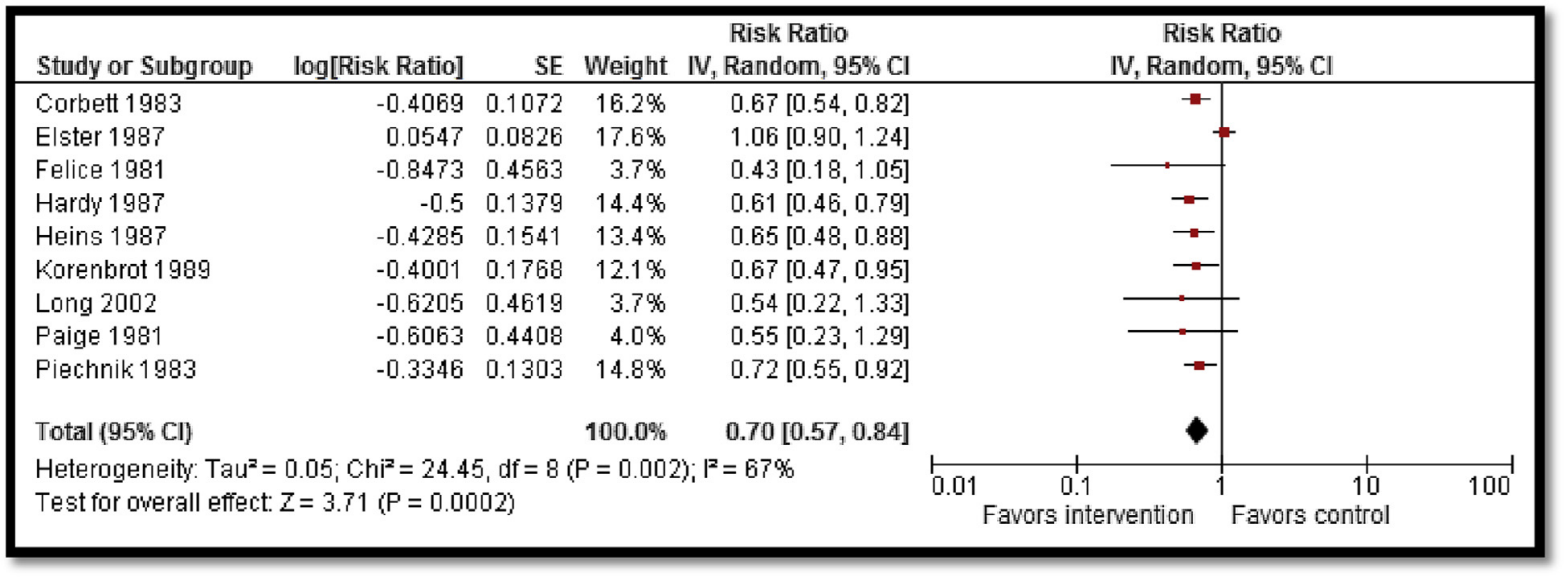
Impact of nutritional interventions for pregnant women on low birth weight. IV = inverse variance; SE = standard error.

A total of 16 studies were included outlining interventions intended to modify maternal diet and reduce adverse maternal and perinatal outcomes. The study participants were low-income, pregnant adolescents from prenatal clinics in urban areas in Chile, Ecuador, United States of America, or Canada, between the ages of 13 and 20 years. All included studies were clinic based except for one school-based [72] and one community-based [69] study. The intervention commenced between 20 and 27 weeks of gestation and continued until delivery. The intervention strategies mainly involved provision of micronutrient supplementation such as calcium and zinc, in addition to the routine iron folic acid supplementation to adolescent mothers or engaging them in nutritional education sessions to enable them to improve nutritional intake. Long-term nutritional counseling was frequently employed whereby pregnant adolescents would have access to a nutritionist whom they would consult as part of antenatal care. Pooled data from moderate- to low-quality evidence suggested a statistically significant improvement in mean birth weight (SMD: .25; 95% CI: .08–.41; Figure 3), reduced LBW (birth weight < 2500 g; RR: .70; 95% CI: .57–.84; Figure 4), and preterm birth (before 37 weeks; RR: .73; 95% CI: .57–.95). These results must be interpreted with caution due to high heterogeneity and very small impact.

### Promoting healthy nutrition and preventing obesity

**Figure 5:**
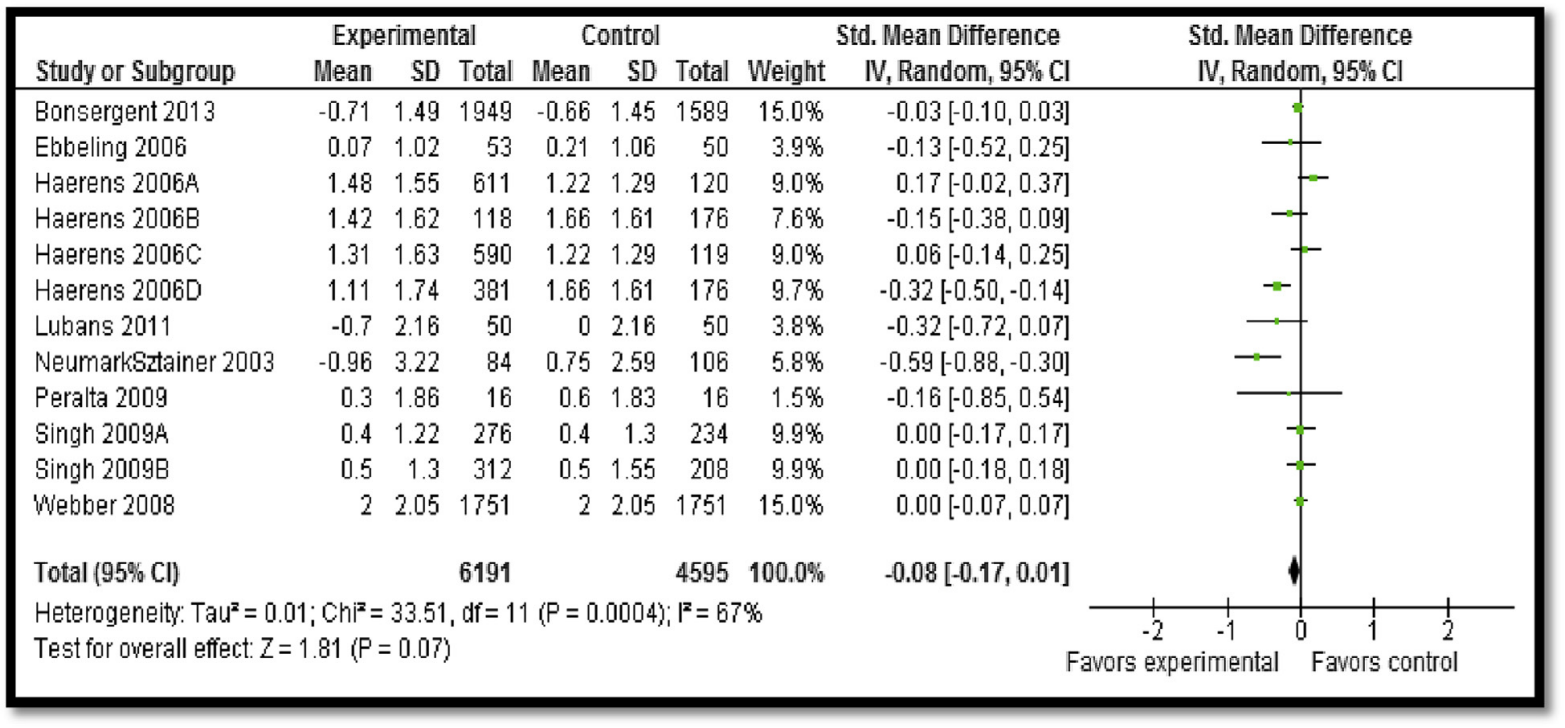
Impact of interventions to prevent obesity on mean change in body mass index. IV = inverse variance; SD = standard deviation.

We updated the existing Cochrane review on promotion of healthy nutrition and preventing obesity by Waters et al. [29] for the age group 11–19 years with an assessment of the methodological quality of systematic reviews rating of 11 for the update. A total of 10 studies (five from the existing review + five new studies) from HICs were included. Overall, the impact on BMI was marginally significant (SMD: −.08; 95% CI: −.17 to .01; Figure 5). Further subgroup analysis revealed that physical activity or dietary control alone did not have any significant impact on BMI reduction while school-based delivery strategies were found to be more effective than interventions in noneducational settings.

## Discussion

Our review suggests that micronutrient supplementation among adolescents can significantly decrease the prevalence of anemia in this age group with school-based supplementation having significant impact while evidence from community-based studies was found to be underpowered. It must be noted, however, that most of these studies were centered on female adolescents and did not take into account the male adolescent population hence limiting the generalizability of the intervention. Most studies on adolescent micronutrient supplementation were conducted in LMICs making the findings context specific and relevant since these settings bear most of the global burden of undernutrition and micronutrient deficiencies. The impact of individual micronutrients and gender-specific impacts could not be segregated. Furthermore, included studies targeted overlapping age groups among the adolescent population that might lead to variations in the outcome effect. However, these findings should be interpreted with caution due to high heterogeneity.

Interventions to improve nutritional status of pregnant adolescents significantly improved neonatal birth weight, decreased LBW, and preterm birth. However, there were insufficient data to evaluate the impact on perinatal, maternal, and neonatal mortality. However, these findings should be interpreted with caution due to high heterogeneity. These findings have limited generalizability since all the findings were from HICs. There is a need to implement the proven interventions in LMIC settings with a higher burden of undernutrition and food insecurity. A focus on adolescent girls' nutrition is important not only to improve the health status of women but also to ensure optimal fetal growth and development to prevent the vicious cycle of intergenerational transmission of undernutrition. Further studies evaluating safety and potential long-term impact of such interventions and cost-effectiveness of these strategies are needed.

Our review suggests that interventions to promote nutrition and prevent obesity can marginally reduce BMI. Evidence from the interventions to prevent obesity mostly comes from HICs hence limiting the generalizability of findings to HIC settings only. With the increasing trend of childhood obesity in LMICs, there is a need for future studies on obesity prevention in LMIC settings [77], [78], [79]. Furthermore, these countries have much higher rates of LBW babies and stunting, and a consequent higher risk of adulthood obesity. There is strong evidence to suggest that once the adolescent is obese, it may be difficult to reverse, with obesity continuing through adulthood, strengthening the case for primary prevention and specific focus on LMICs [80], [81].

Existing reviews on nutrition promotion and obesity prevention have overlapping age groups and include children, adolescents, and youth. Our findings are in concordance with other reviews that suggest beneficial impacts of programs that combine the promotion of healthy dietary habits and physical activity on preventing obesity in children and adolescents, especially school-based programs [82], [83]. Furthermore, evidence exists that a combination of interventions including nutrition, physical activity, knowledge, attitudes, or health-related behaviors has the potential to reduce the risk factors associated with obesity among preadolescent girls (7–11 years), although the sustainability of the effects of such interventions is less clear [84]. Some studies also highlighted important barriers to increasing physical activity among girls including lack of suitable places, resources, and social support for physical activity hence limiting compliance with the intervention program [29].

Limitations of our review include high heterogeneity, lack of data from LMICs, and lack of data to conduct gender-specific subgroup analysis. There is sufficient evidence suggesting the importance of adolescent nutrition interventions and its impact on improved adolescent nutrition and birth outcomes. Countries should now specifically focus on this age group and design programs accordingly with a greater focus on reaching out to this vital segment of the population through schools. There is a need to adopt multisectoral approach in targeting the adolescent age group involving schools and communities through policies and programs to improve adolescent nutrition. Future studies, especially obesity related, should focus on LMICs and underprivileged populations in HIC settings to have maximum impact on improving adolescent nutrition status and reducing adverse neonatal outcomes in these settings. There is also a need to investigate the association of improved adolescent nutrition with improved cognition and future productivity.

## Figures and Tables

**Table 1:** Characteristics of included studies

Author, year	Study design	Country	Setting	Intervention	Target population	Outcome assessed
Micronutrient supplementation						
Agarwal et al., 2003 [30]	Quasi	India	Government school	Iron and folic acid	11- to 18-year-old girls	Hemoglobin
Ahmed et al., 2005 [31]	Before–after	Bangladesh	School	Twice weekly IFA or MMN + IFA	14- to 18-year-old anemic girls	Anemia
Ahmed et al., 2010 [32]	RCT	Bangladesh	School	IFA, MMN	11- to 17-year-old anemic girls	Hemoglobin, serum ferritin, serum vitamin A
Angeles-Agdeppa et al., 1997 [33]	RCT	Indonesia	Senior government school	IFA, vitamin C, retinol	14- to 18-year-old adolescents	Anemia, low ferritin, low retinol
Bruner et al., 1996 [34]	RCT	U.S.A.	Catholic schools	Iron	13- to 18-year-old girls	Hemoglobin, serum ferritin
Chiplonkar and Kawade, 2012 [35]	Quasi	India	School	Zn supplement, diet supplement with Zn and MMN	10- to 16-year-old girls	Hemoglobin
Clark et al., 1999 [36]	RCT	U.K.	School	Zinc supplements	11- to 14-year-old girls	Serum zinc
Deshmukh et al., 2008 [37]	Before–after	India	Community based	IFA	14- to 18-year-old girls	Anemia, hemoglobin levels
Dongre et al., 2011 [38]	Before–after	India	Community based	IFA	12- to 19-year-old girls	Anemia
Eftekhari et al., 2006 [39]	RCT	Iran	School based	Iron and iodine	High-school girls	Hemoglobin, serum ferritin
Februhartanty et al., 2002 [40]	Quasi	Indonesia	Community based	IFA	11- to 15-year-old girls	Hemoglobin, serum ferritin
Friis et al., 2003 [41]	RCT	Kenya	School	MMN	9- to 18-year-old children	Hemoglobin
Goyle and Prakash, 2011 [42]	Before–after	India	School	IFA, vitamin A, vitamin C, iodine	11- to 16-year-old girls	Hemoglobin, serum iron
Guillemant et al., 2011 [43]	Quasi	France	Jockey training school	Vitamin D	16- to 18-year-old males	Serum vitamin D, serum PTH
Hettiarachchi et al., 2008 [44]	RCT	Sri Lanka	School	Iron, zinc	12- to 15-year-old children	Hemoglobin, serum ferritin, serum zinc
Horjus, 2005 [76]	Before–after	Mozambique	School	IFA	11- to 18-year-old girls	Hemoglobin, anemia
Ilich-Ernst et al., 1998 [45]	RCT	U.S.A.	Community based	Calcium supplements	8- to 14-year-old girls	Hemoglobin
Kanani and Poojara, 2000 [46]	Quasi	India	Community based	IFA	10- to 18-year-old girls	Hemoglobin
Khadilkar et al., 2010 [47]	RCT	India	School	Vitamin D and calcium	14- to 15-year-old girls	Serum vitamin D, serum PTH
Kianfar et al., 2000 [48]	RCT	Iran	School	Iron	High-school girls	Anemia
Kotecha et al., 2009 [49]	Before–after	India	School	IFA	14- to 17-year-old girls	Anemia, low serum
Lehtonen-Veromaa et al., 2002 [50]	Quasi	Finland	Local club and school based	Vitamin D	9- to 15-year-old girls	Serum vitamin D
Mann et al., 2002 [51]	Before–after	India	University	Iron and energy supplements	16- to 20-year-olds	Hemoglobin, serum iron
Mwaniki et al., 2002 [52]	RCT	Kenya	School	MMN, antihelminthics	9- to 18-year-olds	Serum retinol
Rousham et al., 2013 [53]	RCT	Pakistan	School	Iron	5- to 17-year-olds	Anemia
Sen and Kanani, 2009 [54]	Quasi	India	School	IFA	9- to 13-year-old girls	Hemoglobin
Shah and Gupta, 2002 [55]	RCT	Nepal	School	IFA	11- to 18-year-old girls	Anemia
Soekarjo et al., 2004 [56]	Before–after	Indonesia	School	IFA, vitamin A	12- to 15-year-old children	Hemoglobin, anemia, low serum retinol
Tee et al., 1999 [57]	RCT	Malaysia	School	IFA	12- to 17-year-old girls	Anemia
Viljakainen et al., 2006 [58]	RCT	Finland	School	Vitamin D	11- to 12-year-old girls	Serum vitamin D, serum PTH
Yusoff et al., 2012 [59]	RCT	Malaysia	School	IFA, vitamin C	16- to 17-year-old children	Hemoglobin
Nutrition in pregnant adolescents						
Chan et al., 2006 [60]	RCT	U.S.A.	Clinic	Orange juice fortified with calcium	Pregnant adolescents ages 15–17 years	Serum electrolyte values, weight, height, blood pressure, and 2-day dietary record
Cherry et al., 1993 [61]	RCT	U.S.A.	Clinic	Zinc supplementation	Pregnant adolescents	Incidence of low birth weight
Corbett and Burst, 1983 [62]	Quasi	U.S.A.	Clinic	Higgins Nutrition Program: consists of an assessment of each pregnant adolescent's risk profile for adverse pregnancy outcomes and an individualized nutritional rehabilitation program based on that profile	Pregnant adolescents	Incidence of low birth weight
Dubois et al., 1997 [63]	Quasi	Canada	Clinic	Higgins Nutrition Program: consists of an assessment of each pregnant adolescent's risk profile for adverse pregnancy outcomes and an individualized nutritional rehabilitation program based on that profile	Pregnant adolescents	Incidence of low birth weight, preterm delivery, and perinatal mortality
Elster et al., 1987 [64]	Quasi	U.S.A.	Clinic	Medical, psychosocial, and nutritional services to pregnant adolescents	Pregnant adolescents younger than 18 years	Incidence of low birth weight and preterm delivery
Felice et al., 1981 [65]	Quasi	U.S.A.	Clinic	Intensive nutritional, psychosocial, and medical intervention and optimal obstetric care.	Pregnant adolescents younger than 15 years	Incidence of low birth weight
Hardy et al., 1987 [66]	Quasi	U.S.A.	Clinic	Nutritional education, group discussions, and psychosocial support	Pregnant adolescents younger than 18 years	Incidence of low birth weight, preterm delivery, and perinatal mortality
Heins et al., 1987 [67]	Quasi	U.S.A.	Clinic	Resource Mother Program: Each resource mother is assigned to a pregnant teenage primigravida and serves as part of her support system throughout pregnancy and until the infant's first birthday.	Pregnant adolescents younger than 19 years	Incidence of low birth weight and perinatal mortality
Hun et al., 2002 [68]	Quasi	U.S.A.	Clinic	Have a Healthy Baby nutrition education program	Pregnant adolescents 14–19 years	Mean birth weight
Korenbrot et al., 1989 [69]	Quasi	U.S.A.	Community	Teenage Pregnancy and Parenting Program	Pregnant adolescents younger than 18 years	Incidence of low birth weight
Long et al., 2002 [70]	Quasi	U.S.A.	Clinic	Supplemental Nutrition Program	Pregnant adolescents	Nutrition knowledge, diet quality, and infant birth weight
Meier et al., 2002 [71]	RCT	U.S.A.	Clinic	Iron supplement	Pregnant adolescents 15–18 years	Birth weight, gestational age, and iron deficiency anemia
Paige et al., 1981 [72]	Quasi	U.S.A.	School	Nutritional supplement	Pregnant adolescents	Mean birth weight
Piechnik and Corbett, 1983 [73]	Quasi	U.S.A.	Clinic	Prenatal screening, patient education, psychosocial evaluation and counseling, nutritional assessment and counseling, intrapartum care, and postpartum follow-up	Pregnant adolescents 12–17 years	Incidence of low birth weight, anemia, and pre-eclampsia
Silva et al., 1993 [74]	Quasi	Portugal	Clinic	Specialized prenatal care	Pregnant adolescents younger than 18 years	Incidence of low birth weight and preterm delivery
Smoke and Grace, 1988 [75]	Quasi	U.S.A.	Clinic	Specialized education program	Pregnant adolescents younger than 18 years	Incidence of low birth weight, preterm delivery, and pregnancy complications

IFA = iron folic acid; MMN = multiple micronutrients; PTH = parathyroid hormone; RCT = randomized controlled trial.

**Table 2:** Summary of findings for the effect of micronutrient supplementation

Quality assessment						Summary of findings		
Number of studies	Design	Limitations	Consistency	Directness		Number of participants		RR (95% CI)
				Generalizability to population of interest	Generalizability to intervention of interest	Intervention	Control	
Anemia: moderate^a^ outcome specific quality of evidence								
11	RCT/quasi	Eight studies had unclear allocation concealment and sequence generation	Two studies showed significant improvement Considerable heterogeneity, *I*^2^ = 72%	All interventions targeted adolescents from both developing and developed countries. Most of the studies involved females only	Majority of the studies involved diet, exercise and behavior change for lifestyle modification, and micronutrient supplementation	6,350	5,511	.69 (.62–.76); (I^2^: 72%)

CI = confidence interval; RCT = randomized controlled trial; RR = relative risk.

a Downgraded for study design and heterogeneity.

**Table 3:** Summary of findings for the effect of nutrition interventions for pregnant adolescents

Quality assessment						Summary of findings		
Number of studies	Design	Limitations	Consistency	Directness		Number of events		RR/SMD (95% CI)
				Generalizability to population of interest	Generalizability to intervention of interest	Intervention	Control	
Mean birth weight: low^a^ outcome-specific quality of evidence								
8	RCT/quasi	Six studies not randomized, selective reporting of outcomes in one study	Only one study suggests benefit Moderate heterogeneity, *I*^2^ = 50%	All studies targeted pregnant adolescents	Interventions included nutritional supplementation and counseling	1,634	1,513	.25 (.08–.41)
Low birth weight (<2,500 g): low^a^ outcome-specific quality of evidence								
9	Quasi	None of the studies were randomized	Five studies suggest benefit Considerable heterogeneity, *I*^2^ = 67%	All studies targeted pregnant adolescents	Interventions included nutritional supplementation and counseling	416	1,011	.70 (.57–.84)
Serum calcium: moderate^a^ outcome-specific quality of evidence								
2	RCT	Selective reporting of outcomes in both studies	No study suggests benefit Low heterogeneity, *I*^2^ = 33%	All studies targeted pregnant adolescents	Interventions included nutritional supplementation and counseling	49	46	−.17 (−.58 to .23)
Preterm birth (before 37 weeks): low^a^ outcome-specific quality of evidence								
2	RCT/quasi	One study not randomized, selective reporting of outcomes in one study	One study suggests benefit Considerable heterogeneity, *I*^2^ = 74%	All studies targeted pregnant adolescents	Interventions included nutritional supplementation and counseling	294	569	.73 (.57–.95)
Iron deficiency anemia: low^a^ outcome-specific quality of evidence								
1	RCT	Selective reporting of outcomes in one study	Only one study	All studies targeted pregnant adolescents	Interventions included nutritional supplementation and counseling	4	10	.34 (.13–.89)

CI = confidence interval; RCT = randomized controlled trial; RR = relative risk; SMD = standard mean difference.

a Downgraded for study design and heterogeneity.

**Table 4:** Summary of findings for the effect of interventions to promote healthy nutrition and preventing obesity

Quality assessment							Summary of findings		
Number of studies	Design	Limitations	Consistency	Directness			Number of participants		MD (95% CI)
				Generalizability to population of interest		Generalizability to intervention of interest	Intervention	Control	
Mean change in BMI: moderate^a^ outcome-specific quality of evidence									
10	RCT	Incomplete reporting of outcomes in three studies	Three studies showed significant improvement Considerable heterogeneity, *I*^2^ = 67%	All studies targeted adolescents	Interventions included diet changes, educations programs, and school-based physical activity programs.		6,191	4,595	−.08 (−.17 to .01)

CI = confidence interval; BMI = body mass index; MD = mean difference; RCT = randomized controlled trial.

a Downgraded for heterogeneity.
